# *“We need them, and they need us*”—Registered nurses’ experiences of leading home care workers caring for dying individuals in their last days of life: A content analysis study

**DOI:** 10.1177/26323524251359677

**Published:** 2025-07-24

**Authors:** Laura Tolboom, Ulla Näppä, Lisbeth Kristiansen

**Affiliations:** 1 Mid Sweden University, Sundsvall, Sweden

**Keywords:** content analysis, focus groups, home care, nursing leadership, palliative care

## Abstract

**Background:**

A mayority of individuals suffering from life-threatening diseases prefer a home death. Registered nurses (RNs) in home healthcare (HHC) play a crucial role in providing this care, as they are responsible for the caregiving process by leading home care workers (HCWs) who provide bedside care, constituting a complex collaboration. However, the research available on this topic, with a focus on nurse leadership, is rather limited.

**Objective:**

This study aimed to explore RNs’ experiences of leading HCWs who were caring for dying individuals in the last days of life.

**Design:**

A qualitative, descriptive, and inductive design was utilized with the help of focus group interviews (FGIs), which involved interviewing 20 RNs employed in HHC in northern Sweden. The FGIs were then analyzed using qualitative content analysis.

**Results:**

The RNs found that a solid working relationship between themselves and HCWs is important to ensure high-quality care in the last days of a patient’s life. They aimed to be available to HCWs and guide them on how to anticipate the dying process and felt responsible for supporting them, often putting their own needs last. The RNs longed for support and guidance themselves while developing their teams. They led HCWs in their development, emphasizing that care in the last days of life was specifically multifaceted, complex, and demanding. Language barriers, organizational challenges, and unclear delineations of leadership responsibilities complicated RNs’ leadership in relation to HCWs.

**Conclusion:**

The RNs favored relational leadership styles, but they faced numerous challenges that varied between urban and rural areas. Moreover, the RNs led by example in dealing with existential feelings, providing care, ensuring symptom management, and fostering communication and teamwork. Through their leadership, marked by compassion and empowerment, they aimed to enhance the quality of care and nurture a supportive network essential for navigating care in patients’ last days of life.

## Introduction

When dying individuals prefer home-based palliative care, they are cared for by various professionals, such as registered nurses (RNs) and home care workers (HCWs), who collaborate to deliver this care.^1^ Teamwork in home healthcare (HHC) is characterized by contextual challenges, such as lone working practice.^2^ While HHC in Sweden is provided by municipalities as a comprehensive service,^3^ RNs and HCWs typically have separate managers and gather in separate nursing facilities.^4^ Notably, HHC in Sweden is structured and regulated by law^5,6^ (Table 1).

**Table 1.** Home care structure, personnel, and legislation (Swedish nursing titles were adapted to increase international readability).

Organizational responsibility	Workforce	Profession	Education level	Nursing licensure	Law
Municipality (under care agreement with county)	Nurses	SNs	Master’s degree in nursing	Yes	The Health and Medical Services Act
		RNs	Bachelor’s degree in nursing	Yes	
	Home care workers	LPNs	Vocational degree in nursing	No, but in Sweden, LPN is a protected professional nursing title	Social Services Act; exceptions are made for tasks within the Health and Medical Services Act under the supervision of RNs/SNs
		NAs	No formal training required	No	Social Services Act

LPNs, licensed practical nurses; NAs, nursing assistants; RNs, registered nurses; SNs, specialist nurses.

RNs in Swedish HHC have extensive responsibilities in overseeing the care process, which has become increasingly complex and demanding due to patient comorbidity and out-of-hospital care.^7,8^ RNs in HHC possess high levels of independence, maturity, skills, and knowledge required for working in HHC in general,^9^ and they often have extensive levels of palliative care experience and competence.^10^ RNs collaborate with HCWs, who offer a broad range of domestic and personal services,^11^ including palliative care.^12^ In recent decades, Swedish HCWs have been assigned more complex medical tasks due to deinstitutionalization and new public management.^13^ HCWs in Sweden can be licensed practical nurses (LPNs) or nursing assistants (NAs). LPNs have to complete a basic nursing program in bedside care, while NAs commonly lack formal nursing training.^14,15^

HCWs provide home-based palliative care with minimal supervision or support.^2,12^ Despite their importance in maintaining this care, the available research on what practical and emotional support is available to them is remarkably sparse.^16,17^ Home care organizations often face challenges such as poor organizational culture, inadequate infrastructure, and a lack of cohesive mission and vision.^18^ HCWs commonly experience minimal support even though their workload has increased and their autonomy has decreased in recent times.^13^ In addition, the HCW workforce struggles with staff shortages that might force caregivers to hire individuals who are not necessarily qualified for the work. In many Western countries, migrants who are willing to do the physically and emotionally demanding home care work for minimal wages are recruited.^19^ In Sweden, 42% of HCWs have a migrant background, and 82% of HCWs are women.^20^

RNs in HHC are meant to support and guide HCWs in palliative care tasks such as sitting vigil, which refers to the act of offering companionship to the dying individual while delivering essential palliative care.^21^ In most countries, it is either a family member or a volunteer who sits vigil.^21–23^ However, in Sweden, professional vigilance for dying individuals is legislated by law to both alleviate the burden on family caregivers and ensure that the patient does not have to die alone.^24^ Professional vigil, which is ordered by RNs and facilitated by HCWs, was a central subject in the focus group interviews (FGIs) conducted for this study. To ensure clarity and international readability, this study refers to the Swedish approach to delivering essential palliative care through professional vigil as “care in the last days of life.” Care in the last days of life specifically pertains to the expected last 2 or 3 days in a patient’s life, which is when the active phase of dying begins.^25^

**Figure 1.**
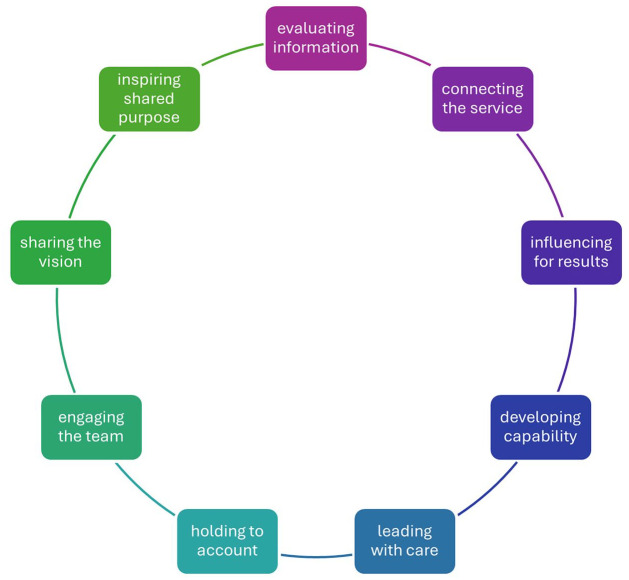
Nine dimensions of nursing leadership according to the NHS Leadership Academy.^28^

It is essential to lead, guide, and support HCWs who are caring in the last days of life due to the complexity and demanding nature of the task.^26^ In recent decades, nursing leadership has shifted away from nurses influencing others in a top-down manner to achieve goals and toward nurses being both leaders and learners in a community-like environment that supports, treats, and cares.^27^ Therefore, nursing leadership can be divided into nine dimensions.^28^ Figure 1 illustrates how leadership was understood in this study.

Benner’s middle range theory—“from novice to expert”^29^—illustrates the various phases of clinical competence development, which involves the dimensions of nursing leadership.^28^ Benner’s theory was later utilized by Quinn,^27^ who emphasized that nurses develop leadership along a continuum, with effective leaders recognizing continuous learning and the importance of teamwork.

### Research problem and aim of the study

**Figure 2.**
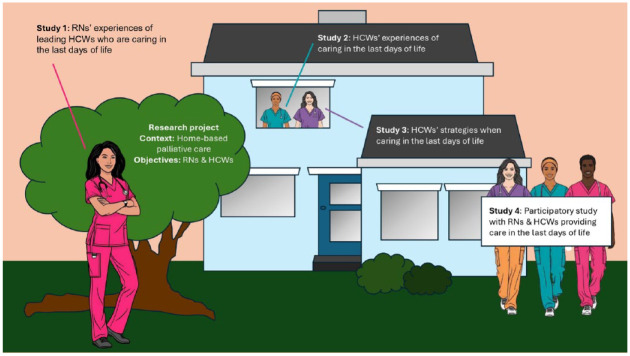
A visual representation of the research project.

Nurses’ experiences of leading HCWs remain underexplored, while HCWs largely rely on RNs’ support when caring for individuals in the last days of life. This care is complex and demanding, thus requiring strong teamwork and nursing leadership. Therefore, collaboration between RNs and HCWs is necessary to sustain home-based care for dying individuals, facilitating the possibility of a home death. Hence, this study aimed to explore RNs’ experiences of leading HCWs as they care for dying individuals in the last days of life. This study is part of a larger research project which, to the best of our knowledge, is the first research project to explore RNs’ and HCWs’ experiences, strategies, and compassion as they provide care in the last days of life. This research project is artfully presented through an illustration to improve clarity and transparency regarding the inclusion and representability of both groups (Figure 2).

## Methods

### Design

This study employed an inductive descriptive approach within the constructivist research paradigm.^30^ The research team assumed that the reality of nursing leadership in this context was constructed socially and experimentally and aimed to explore the meanings of these constructs.

### Participants

In total, 20 RNs were included in the data collection, which was carried out in northern Sweden from June 2024 to November 2024. The participants were identified through purposeful sampling^31^ and selected from 10 municipal HHC teams from 2 counties. Although the participants included both RNs and NSs, they were all referred to as RNs. They all had experience in leading HCWs as they provided care in the last days of life.

The RN managers received written information and provided written consent to recruit from their staff. The RNs received verbal and written (via email) information concerning the study, and they submitted their written consent to participate. The demographic data from the RNs were collected (Table 2) and stored in a personal and password-protected cloud storage approved by Mid Sweden University.

**Table 2.** Variations in participant characteristics.

Participants n	Gender (male, female, other)	RN/NS	Age	Years of work as a nurse	Years of work in home care	Works in rural/urban areas
20	0/20/0	RN 9/NS 11	26–61 (m 42)	2–36 (m 17)	1–24 (m 7)	12/8

m, median; RN, registered nurse; SN, specialist nurse.

### Data collection

The data collection was conducted through five digital FGIs,^32^ led by a moderator and an assistant moderator (Table 3). The FGIs were digitally recorded by the moderator. RNs from different municipalities were purposely grouped in the FGIs, which were conducted in Swedish and lasted between 1 and 2 h, with no RNs withdrawing from the interviews.

**Table 3.** Focus group characteristics.

Characteristics	FGI 1	FGI 2	FGI 3	FGI 4	FGI 5
Participants	4	3	4	6	3
Age	34–44 (m 40)	42–61 (m 58)	32–56 (m 38)	26–61 (m 42)	42–57 (m 45)
Years as RN	4–25 (m 11)	16–34 (m 18)	5–24 (m 10)	2–36 (m 13)	18–36 (m 22)
Years in home care	4–12 (m 4)	5–24 (m 5)	1–20 (m 4)	2–19 (m 8)	8–12 (m 10)
Moderator and assistant moderator	L.T. and U.N.	L.T. and U.N.	L.T. and U.N.	L.T. and L.K.	L.T. and L.K.
Duration FGI	1 h 32 min	1 h 35 min	1 h 47 min	1 h 57 min	1 h 5 min

FGI, focus group interview; m, median; RN, registered nurse.

The moderator guided the conversations, and the assistant moderator supported the moderator by making notes and summarizing the interviews. The moderator used a topic guide containing three main questions (Table 4).

**Table 4.** Three main questions from the topic guide.

What support do you need to provide to HCWs so they can manage care in the last days of life?
What do you, as a nurse, need to be the leader for HCWs that you believe you should be?
How do you perceive the current ability of HCWs to fulfill the task of caring in the last days of life?

HCWs, home care workers.

### Data analysis

The data were analyzed through qualitative content analysis^33,34^ to methodically arrange and interpret content from the FGIs identifying patterns and themes that were pertinent to the research question.^31–33^ Through this process, both explicit and implicit content from the FGIs was interpreted, revealing both the depth and the meaning of the nursing leadership experiences.

The FGIs were transcribed verbatim, after which the text was sorted, abstracted, and interpreted in various phases, involving a nonlinear process where the text was decontextualized and recontextualized.^34,35^ The analysis resulted in codes, (sub)categories, themes, and an overarching theme, revealing both manifest and latent content through the following steps.

Initially, the transcriptions were carefully read by the first author multiple times to grasp the overall meaning. Then, the RNs’ experiences of leading HCWs providing care in the last days of life were extracted from the texts separately for each FGI, constituting the meaning units.^33^ Each meaning unit was then concentrated into condensed meaning units, which contained manifest content about nursing leadership experiences. These condensed meaning units were further condensed by the first author and labeled with a code reflecting the content of the condensed meaning unit.^33^ This way, the manifest key concepts from the FGIs were identified, after which the research team created subcategories by sorting codes based on their similarities and differences and, finally, abstracting and formulating subcategories (Table 5).

**Table 5.** One example of the process of analysis.

Meaning unit	Condensed meaning unit	Code	Subcategory
However, I think one must be comfortable in being able to deal with death. It does not matter if you have worked for one year or 50 years; you must be able to feel secure about death and handle it. [RN x]	You must be comfortable dealing with dying and death, regardless of how experienced you are	Dealing with death and dying	Exposure to death

The subcategories were grouped together and interpreted to formulate categories, revealing a higher level of abstraction. These categories were, in turn, interpreted and paired to form themes and an overarching theme that captured the latent content of the data. The analysis was thoroughly discussed by the research team to ensure that it accurately reflected the essence communicated in the FGIs. The results were translated into English after the final step of the analysis.

### Ethical approval and considerations

This study was approved by the Swedish Ethical Review Authority (no. 2024-02692-01). All data in this study were handled carefully by the research team in accordance with the regulations of the General Data Protection Regulation (GDPR) legislation for the protection of sensitive personal data.^36^

The RNs were informed at the start of the data collection process that withdrawing their participation could pose challenges due to the nature of FGIs.^32^ They were also informed that participating in this study could evoke negative feelings. Considering this, the research team had anticipated providing additional support after the FGIs if necessary. This additional support was indeed executed, with the research team reaching out through email. Moreover, FGIs also pose ethical challenges, such as those related to the management of conversations and the protection of sensitive information.^37^ Confidentiality concerns,^37^ such as sharing stories outside the FGI, were communicated with the RNs. Precautions were taken to minimize risks before, during, and after the interviews^36^ by keeping participation confidential from third parties and emphasizing mutual respect within the groups.

This study followed the Consolidated criteria for reporting qualitative research (COREQ)-questionnaire for interviews and focus groups^38^ (Supplemental Appendix 1).

## Results

**Figure 3.**
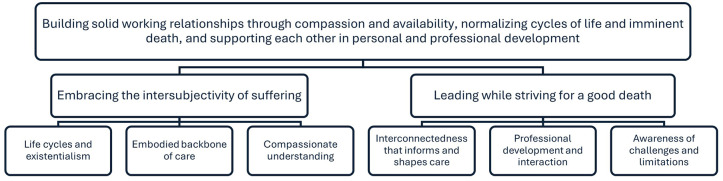
The overarching theme, two themes and six categories.

The RNs’ experiences of leading HCWs were abstracted into an overarching theme built on two themes, each of which had three underlying categories (Figure 3).

### Overarching theme

The overarching theme was “Building solid working relationships through compassion and availability, normalizing cycles of life and imminent death, and supporting each other in personal and professional development.” The RNs strived to build solid working relationships with HCWs through compassion and availability. They expressed feeling responsible for supporting HCWs manage their feelings by emphasizing that death was a natural process. The RNs aimed to guide and support HCWs to enhance their personal and professional development, as the RNs found caring in the last days of life to be specifically multifaceted, complex, and demanding.

#### Theme 1: Embracing the intersubjectivity of suffering

Embracing the intersubjectivity of suffering refers to the shared experiences between RNs and HCWs when caring for dying individuals, transcending individual experiences. Exposure to the process of dying evoked intense feelings about mortality and fueled reflection on life and existentialism among the RNs. This involved the RNs’ personal predispositions and their ability to endure a working environment where everyone relied on them, regardless of their state of mind and resources. Therefore, RNs were perceived as the backbone of care. Additionally, the intersubjectivity of suffering fostered compassionate understanding between RNs and HCWs, as both needed closeness and availability.

##### Category 1: Life cycles and existentialism

The RNs engaged in conversations with HCWs about death and dying, stating their belief that if they approached this topic in a natural manner, this behavior would be adopted by HCWs. Sharing experiences of managing death and dying by senior colleagues was highly valued by the RNs, as they believed that it assisted less experienced RNs in gaining professional attitudes toward dying, which helped them lead and support HCWs while caring in the last days of life:

I think we need to be open about death, you know. We have to . . . it’s not something that is shameful, embarrassing, terrible, or difficult. It’s a natural process. I believe we need to . . . talk a lot about death and try to help HCWs feel secure in the knowledge that people die. (RN2); To downplay death . . . we have a colleague here who has worked for several years in specialized palliative care and . . . a younger colleague came in, clearly thinking, oh my . . . what if this patient we are going to visit dies? Then, the older colleague said, well, that’s great, isn’t it? That’s what we’ve been waiting for. (RN1); We don’t truly know what the HCWs carry with them. It could be that they have experiences from . . . their private life, which can also complicate things . . . at work. (RN2; FGI 1).

The RNs mentioned feeling that HCWs struggled to understand their roles and responsibilities when caring in the last days of life, asking the RN to lead them. However, the RNs acknowledged that death and dying evoked intense feelings and aimed to provide additional support to HCWs who struggled with these feelings. The RNs also felt that a lack of support was particularly evident during palliative care, especially when caring for younger individuals. Reflecting with HCWs on care in the last days of life fostered a greater understanding of this care. This was challenging when the RNs and HCWs did not have similar perspectives on this care, and frustrating if HCWs did not adhere to the instructions provided by the RNs. In complex care situations, the RNs preferred to provide bedside care tasks themselves instead of assigning them to a HCW, and some HCWs and RNs feared harming the dying individual.

##### Category 2: Embodied backbone of care

The RNs emphasized the importance of feeling secure and supported themselves while leading HCWs. When they worked with competent HCWs, their sense of security improved. In such situations, they felt prepared and could anticipate changes in the dying individual’s health status while supporting and guiding the HCWs. They highlighted their own need for guidance and support to provide sufficient nursing leadership to HCWs, as many RNs felt that they received inadequate support and guidance themselves:

No, there’s no support to us, it feels like we just have to keep working on. (RN15); Oh yes, and it can be tough situations sometimes. (RN14); No, no . . . they’re all human . . . the HCWs. It’s just that . . . and we are too. We are not . . . superhumans, we are just people who must handle it as well. (RN15; FGI 4)

Initiatives were undertaken by the RNs to improve collaboration between HHC and primary care physicians to enrich their supportive network. Occasionally, they lacked support from other RNs due to differing palliative care perspectives, which complicated leading and caring in the last days of life.

HCW managers played a crucial role in enabling RNs to be effective leaders for HCWs when delivering care in the last days of life. Some of the managers worked closely with HCWs, providing them with low-threshold support and guidance. The RNs expressed their belief that it was advantageous when these managers had previously worked in a caring profession, as they would inherently understand the needs and dynamics of palliative care situations. The RNs thought HCW managers needed palliative care competence and to take responsibility for the caretaking process. Collaboration between RNs and HCW managers was vital to secure sufficient resources for HCWs; however, this collaboration was challenged by the lack of a clear delineation of leadership responsibilities between HCW managers and RNs:

No, I sometimes feel like it’s as if we take on everything ourselves, but sometimes you must think that, yes, sometimes I feel like we should handle everything. I don’t know, but a lot should be put back on. . . on the manager of the HCWs as well, but if the basic care isn’t managed in the end, they [the patients] will get sick, and then . . . then it ends up being on us anyway. (RN10); It feels like it should be . . . should be more . . . organized or however you want to put it, that it should already be part of our tasks. Maybe not just like this “good [last days of life] care,” but that it should be a bit more specified that “this is included in. . .”—like the responsibility that the manager has and that the RN has and that the manager and the RN should collaborate on this. (RN8); The HCWs are not my personnel . . . They are not my employees. We’re the same group but also not. So, it gets a bit complicated, the hierarchy that is . . . becomes complicated. You don’t know if you’re allowed or not. (RN11; FGI 3)

HCW managers were perceived by the RNs as bearing a heavy workload, which impacted the managers’ ability to lead HCWs themselves. Consequently, the RNs had to step in as backup for managers to lead and support HCWs despite their own heavy workloads. While leading HCWs was not an explicitly defined task for the RNs, they were expected to provide support and guidance at any time and place. The RNs participated in team development initiatives, but owing to limited or withdrawn resources, promising palliative care projects were commonly paused or terminated.

##### Category 3: Compassionate understanding

The RNs expressed a need for proximity to HCWs while being empathic and compassionate toward them. They valued experiences that involved delivering compassionate care along with HCWs. Closeness and a sense of security were enhanced through teamwork involving HCWs and, specifically, RNs’ leadership. The RNs emphasized the importance of being flexible and available to HCWs. The positive teamwork experiences were particularly evident in rural areas, as the RNs in these areas were often acquainted with the HCWs, and they believed that familiarity enhanced care quality and tailored support, knowing who was providing care in the last days of life. Being together with RNs alleviated HCWs’ feelings of loneliness and increased their confidence in delivering care:

But then we also try to be physically present during the day, not for long periods, but just to show up and talk and demonstrate things as much as possible. Even during nights, one nurse usually goes, even if it’s not their assigned patient . . . I can still go and help and support, but it won’t be for long periods; it’s just about being available. (RN7); It is so important . . . everything . . . especially in the dying phase, where it feels like everything you do matters . . . even the smallest gesture or what you have said makes you reflect on yourself to ensure that nothing goes wrong. (RN6; FGI 2)

Moreover, in rural areas, sharing space and having each other in proximity allowed meetings that fostered collaboration between RNs and HCWs, allowing RNs to properly lead the HCWs. The RNs desired to engage with HCWs, both individually and in groups, to discuss care in the last days of life. Conversely, the RNs in urban areas felt a distance from HCWs, leading to infrequent or absent meetings, unfamiliarity with HCWs, and diminished control. They attempted to connect with the HCWs but had difficulty reaching out. The HCWs desired closer contact and collaboration when they felt uncertain about caring in the last days of life. The fixed minute-to-minute schedules of HCWs complicated this, along with the travel times in large municipalities—a shared concern in both rural and urban areas.

#### Theme 2: Leading while striving for a good death

Leading while striving for a good death for the patients involved the RNs’ efforts to create the necessary conditions for care in the last days of life. This was a complex process, requiring the RNs to advocate for the patients’ needs, lead the HCWs, and manage practical nursing tasks. Managing delegation reflected the interconnectedness between the RNs and HCWs that shaped the care process. This transformed the caregiving relationships, emphasizing the importance of shared understanding and responsiveness in providing care during the last days of life. The team development and collaboration required for providing care in the last days of life were challenging in urban areas due to a shortage of experienced HCWs to meet the needs of many patients. The RNs were aware of the many challenges and limitations that hindered their ability to lead, support, and guide HCWs.

##### Category 4: Interconnectedness that informs and shapes care

The RNs emphasized the importance of assigning HCWs with nursing tasks, ensuring continuous alleviation of symptoms. Practical solutions for symptom relief, such as delegation, were essential in large municipalities to reduce travel time. Fruitful collaboration with competent HCWs resulted in peaceful last days of life and contributed to a good death. Moreover, the RNs felt secure and in control when the HCWs independently delivered good palliative care, which alleviated the burden that the RNs felt in leading and supporting HCWs:

In our municipality, we delegate . . . to give subcutaneous injections. . . and that has made things easier and . . . as I see it, it’s also a very quick help they receive, I mean . . . sometimes we have over an hour travel time, and it would never work if we had to visit everyone who needed injections and . . . it works fantastically well . . . No, and it’s like it can never go wrong, they always call before they give injections so that . . . no, it works fantastically well for us. (RN19); We don’t have that. (RN18); We can delegate injections as well. But here, it’s more those who live in rural areas that we delegate to . . . here in the city, we have short distances, so we usually give the injections ourselves. And there, I also experience that there is a difference between the staff who work in rural areas and those who work . . . more in the city, in that they are more secure and often have more experience, yes, but like, in home care. (RN20; FGI 5)

The RNs reported that not all HCWs were suitable for delegation, and when unable to delegate sufficiently, the RNs had to prioritize their own visits as they ran out of time for all assigned patients, leading to stress and diminished control. Moreover, the RNs also expressed their worries about delegating tasks to inexperienced HCWs and being unable to deliver care themselves, which left them feeling insecure and contemplating that care in the last days of life was both rewarding and challenging.

When caring for dying individuals, the RNs felt that a steady HCW workforce yielded better care outcomes. Instructing HCWs on bedside care, alongside creating an optimal care environment, was the core responsibility of RNs. The RNs stressed the importance of physicians engaging in conversations with dying individuals to provide appropriate care instructions to HCWs. Further, they advocated designating care in the last days of life (through political decision) as a task requiring nurse delegation, as this would provide quality assurance.

##### Category 5: Professional development and interaction

The RNs working in rural areas generally had positive experiences with HCWs providing care in the last days of life, as some areas only assigned steadily employed and healthcare-educated HCWs to this care. These competent HCWs met the right standards, providing secure care with minimal leadership required from the RNs. It was common in urban areas that RNs had to lead by providing extensive practical guidance to HCWs, who often lacked formal healthcare education, competence, or experience with care in the last days of life. The RNs agreed that these HCWs should preferably not be assigned to this care at all. Experienced and competent HCWs existed in urban areas as well, albeit sparsely, and were often unavailable because of the many elderly individuals with complex care needs:

We try to avoid placing those [HCWs] who have never . . . well, who have no experience with . . . a dying person. We don’t assign them . . . those HCWs to those patients. Instead, we try to take those who are more experienced in . . . in the care . . . the palliative care. And then it becomes a safer care overall, both for the HCWs and for the patient, if they are used to it, and for us as well, because then we also know that they . . . can and know what to do. (RN2); I think that there is this little view. . . that . . . oh . . . they are only sitting vigil. It’s like you just take the first person you can call in . . . but then this person . . . may have never seen such a sick patient or witnessed someone pass away [. . .] I think, if you put a young person there who has never seen anyone . . . you could scare someone for life, and they would never want to set foot in healthcare again. (RN1; FGI 1)

The RNs expressed that HCWs had to be engaged to gain palliative care competence. They led HCWs by providing flexible support and guidance, owing to the great variety within this workforce. Sometimes, they reallocated HCWs to facilitate the dying individuals’ best interests and protect the HCWs. They also felt that tailored leadership toward HCWs was demanding and multifaceted, and they even considered it to be an art.

The development of the team was desired by the RNs, who wished for a stable and small HCW workforce to engage in educational meetings with. They stated that it was their responsibility to educate HCWs about palliative care, as this care was perceived by the RNs as important and sensitive. They also longed for educational meetings themselves to stay informed about the latest developments in palliative care. In some rural areas, there were daily meetings between the RNs and HCWs, but most RNs experienced an insufficient number of meetings with HCWs. They voiced their belief that education alone was insufficient for providing care in the last days of life, emphasizing that social skills and personality were as important as knowledge. The already challenging position of HCWs was considered even worse for those without permanent job positions, particularly concerning resources.

##### Category 6: Awareness of challenges and limitations

The RNs were aware of their own limitations and were grateful to be able to collaborate with specialized palliative care teams, as it provided them some support, thus enabling them to focus on leading HCWs. The collaboration with specialized palliative care teams was primarily between nurses and physicians and did not reach HCWs. Some municipalities had RNs and HCWs who, despite having qualifications in palliative care, were hindered by limited resources from sharing this knowledge and competence.

Language barriers between HCWs and RNs in urban areas impeded effective collaboration when caring in the last days of life. Despite the RNs’ efforts to lead and support HCWs, this care was frequently provided with limited guidance due to time constraints and language barriers, which jeopardized safe care:

So often, those [RNs] who work in rural areas . . . tend to have a larger proportion of staff who are more experienced [. . .] and here in the city, well . . . I mean, many in our [HCW] groups . . . it’s like their first job, and you don’t need a driver’s license. It’s kind of the first thing you take as a part-time job, but not like in the rural areas, you feel that you have more experienced and stable staff there. (RN13); That’s right. (RN14); But that’s how it is, and there is a larger turnover in the urban areas with staff. (RN15); Yes, yes, but it’s an extreme situation. It’s an extreme number of staff turnovers, and it also leads to more problems with language barriers and such. (RN13; FGI 4)

HCWs sometimes felt overwhelmed by the responsibility of supporting both the dying individual and their family. The RNs emphasized that providing general home-based care and specifically home-based palliative care was challenging, and the lack of resources diminished their ability to lead HCWs. The RNs pointed to the diversity of HCWs, noting that not all were equally sensitive in delivering care in the last days of life. Some HCWs reportedly exhibited a lack of compassion or engagement, making it difficult for the RNs to provide meaningful guidance. While some municipalities employed HCWs with extensive experience or training in palliative care, differentiation among these workers was generally minimal.

## Discussion

This study aimed to explore RNs’ experiences of leading HCWs providing care for dying individuals in the last days of life. The RNs reported that adapting nursing leadership to address the needs of HCWs was challenging and complex—to the point that it could be regarded as an art. They aimed to lead by example, acknowledging lifelong learning and the importance of reflection and togetherness. Empowering HCWs was crucial to the RNs because they felt that caring in the last days of life evoked strong existential feelings. Therefore, they strived to be compassionate leaders to the HCWs. However, the RNs themselves had limited possibilities for self-compassion and support. They reminisced about how HHC used to be compared to today’s practice, which suffers from a lack of teamwork, communication issues, and limited resources.

The study aimed to navigate all dimensions of nursing leadership, moving away from a traditional top-down approach and instead focusing on inclusive leadership characterized by compassion, learning, and reflection. Quinn’s writings on nursing leadership^27^ guided the research team through these dimensions, and the research team reflected on systemic inequalities in home care, emphasizing the need for structural change that values all labor.

In this study, the RNs aimed to build and maintain working relationships and strong teams with HCWs through nursing leadership; however, this was made difficult because of restricted resources. This could lead to task-focused leadership, which is associated with low care outcomes and job satisfaction. Previous research has shown that relational nursing leadership improves staff recruitment, retention, and work environments, ultimately leading to increased job satisfaction.^39^ Even RNs themselves benefit from relational leadership styles, focusing on compassion, credibility, and trust while engaging and empowering others with a willingness to learn, as well as engaging with reflection and self-care.^27^

The RNs in this study experienced opportunities and obstacles, noting the overall differences between municipalities and between rural and urban areas. Resources such as access to experienced HCWs and interdisciplinary meetings differed in this study, but common issues such as time constraints and staffing shortages were also identified. Moreover, previous research has also revealed a magnitude of obstacles, such as a growing workload, organizational shifts, and prioritizing cost-effectiveness, that have worsened working conditions in Swedish home care^13^; therefore, there is a need to strengthen (palliative) home care.^40^

Notably, the RNs in this study emphasized that they often placed their own needs last, recognizing this as a long-term concern. Previous research has shown that mental exhaustion is a serious concern within both the HCW workforce^13^ and the RN workforce.^41^ RNs would benefit from self-compassion to cope with the emotional labor of caring in the last days of life. Promoting well-being should be an integrated component of nursing education and the support that RNs receive.^42^

The heterogeneity of the HCW workforce challenged the RNs in this study, requiring their flexibility and versatility in providing tailored leadership. Moreover, language barriers between RNs and HCWs were also found to be common. The RN workforce in Sweden is homogeneous and predominantly female.^43,44^ While the HCW workforce is also predominantly female, there is great variation in other demographics.^13^ Working as a HCW in Sweden is generally perceived as a low-status and entry-level job, which has, together with an aging population, led to significant staff shortages.^13^ These shortages are largely addressed by immigrants,^45^ who are learning the language while working as HCWs. According to the RNs participating in this study, this posed challenges for all parties involved, with no straightforward solutions available—a challenge recognized in other countries.^46,47^ The communication difficulties reflect modern home care dynamics, where language barriers not only hinder practical interactions but also affect the way both HCWs and RNs experience their roles and identities within the spatial context, raising questions about who gets to speak and who remains unheard. They reflect broader societal structures that privilege certain voices while silencing others. Given the frictions between the HCW workforce and resources available in HHC, a radical reorientation toward equality and sustainability is needed, moving away from an overemphasis on cost-effectiveness.^48^ Challenges could be addressed through empowerment and equality of all workers, which would contribute to shaping professional identities and care outcomes.^49^ This could strengthen the status of both RNs and HCWs, as well as the symbolic capital of home care nursing, which has declined in recent years.^50^

This study emphasized the need for inclusivity in home care through relational nursing leadership to foster a cohesive, compassionate, and supportive care environment. It underscored a broader societal obligation to create orientations in home care that recognize and valorize diversity within the caregiving space. Furthermore, it is paving the way for more research in this area, which could benefit palliative care, future policy decisions, or societal change. Research on specific areas of nursing leadership in the last days of life could be beneficial. Studies that illuminate the experiences of HCWs who care for individuals in the last days of life would provide a more complete picture of this care.

## Conclusion

The RNs played a crucial role in leading HCWs, addressing the emotional complexities surrounding death, providing bedside care, ensuring symptom management, and fostering effective communication and teamwork. Their adoption of a relational leadership style, characterized by compassion, credibility, and empowerment, not only enhanced care quality but also nurtured a supportive network essential for navigating the complexities of this care. The efforts of RNs and HCWs are pivotal during home-based care in the last days of life, as they strive to deliver comprehensive and compassionate care amid numerous challenges. While the obstacles they face often vary between urban and rural areas, certain challenges, such as staffing shortages, communication difficulties, heavy workloads, and rigid scheduling, remain prevalent across both areas.

## Methodological considerations

The transferability of this study is limited because it was conducted in northern Sweden, where population density is low,^51^ which could entail a need for different types of nursing leadership within home-based palliative care compared to the economic, spatial, and social aspects of densely populated regions.

Digital FGIs generate a similar experience and have similar outcomes to in-person FGIs.^52^ The digital FGIs allowed RNs to share experiences with colleagues from different municipalities, which was a notable strength of this study. Moreover, the multitude of experiences and discussions shared by the RNs created rich data, which allowed the research team to gain a nuanced image of what nursing leadership when caring in the last days of life could entail.

The RNs found the FGIs valuable and wished for similar peer meetings. The FGIs facilitated the identification of commonalities^53^; however, mixing RNs from different municipalities could lead to feelings of injustice when working conditions between RNs differed. The FGI design requires participants to keep each other’s sensitive information, which was communicated. The RNs might have felt hesitant to share their experiences with strangers; therefore, the research team has actively created space for all participants.

L.T.’s preunderstanding consisted of working in home-based palliative care. Three participants had previously collaborated with L.T. in HHC. After discussing this within the research team and with the participants, it did not lead to exclusion. U.N. and L.K. worked previously in palliative care and psychiatric care, respectively. The research team discussed their preunderstanding throughout the entire process of this study to minimize bias. The data were approached with an openness and expectation of finding new perspectives.

## Supplemental Material

sj-docx-1-pcr-10.1177_26323524251359677 – Supplemental material for “We need them, and they need us”—Registered nurses’ experiences of leading home care workers caring for dying individuals in their last days of life: A content analysis study Supplemental material, sj-docx-1-pcr-10.1177_26323524251359677 for “We need them, and they need us”—Registered nurses’ experiences of leading home care workers caring for dying individuals in their last days of life: A content analysis study by Laura Tolboom, Ulla Näppä and Lisbeth Kristiansen in Palliative Care and Social Practice
