# Supplementary material for: “We need them, and they need us”—Registered nurses’ experiences of leading home care workers caring for dying individuals in their last days of life: A content analysis study
Source: Palliat Care Soc Pract. 2025 Jul 24;19:26323524251359677. doi: 10.1177/26323524251359677 (PMC12301595; doi:10.1177/26323524251359677)
Supplement: sj-docx-1-pcr-10.1177_26323524251359677 – Supplemental material for “We need them, and they need us”—Registered nurses’ experiences of leading home care workers caring for dying individuals in their last days of life: A content analysis study [file sj-docx-1-pcr-10.1177_26323524251359677.docx]

Appendix 1

COREQ: 32-item checklist

| **No** | **Item** | **Guide questions/description** | **Page** |
| --- | --- | --- | --- |
| **Domain 1: Research team and reflexivity** | | |  |
| **Personal Characteristics** | | |  |
| **1.** | Interviewer/facilitator | The focus groups were conducted by LT, UN and LK | 9 |
| **2.** | Credentials | LT – RN MSC, PhD student nursing science  UN – RN MSC, dr., senior researcher in nursing science  LK – RN MCS, professor of nursing science | N/A |
| **3.** | Occupation | All researchers have an occupation at Mid Sweden university | N/A |
| **4.** | Gender | LT, UN, LK – female | N/A |
| **5.** | Experience and training | LT is a doctoral student with an extensive experience in palliative care nursing, specialist nurse in palliative care.  UN and LK are senior researchers, UN is specialised  in oncology and palliative care and LK is specialised in psychiatric care. | N/A |
| **Relationship with participants** | | |  |
| **6.** | Relationship established | Prior to the study, there was some familiarity between LT and some of the participants, because of LT working as a nurse in palliative home care and  meeting RNs in HHC in the region. | 30 |
| **7.** | Participant knowledge of the interviewer | The FGIs started with a short introduction moment. Participants were told some of the background of  the study and the researchers and aim of the study. | 9 |
| **8.** | Interviewer characteristics | The researchers shared some of their reasons and interests for conducting the study. | 30 |
| **Domain 2: study design** | | |  |
| **Theoretical framework** | | |  |
| **9.** | Methodological orientation and Theory |  | 10 |
| **Participant selection** | | |  |
| **10.** | Sampling |  | 8 |
| **11.** | Method of approach |  | 9 |
| **12.** | Sample size |  | 8 |
| **13.** | Non-participation |  | 9 |
| **Setting** | | |  |
| **14.** | Setting of data collection |  | 9 |
| **15.** | Presence of non- participants | There were no other individuals present during the FGIs. | N/A |

| **16.** | Description of sample |  | 9 |
| --- | --- | --- | --- |
| **Data collection** | | |  |
| **17.** | Interview guide |  | 10 |
| **18.** | Repeat interviews | No FGIs were repeated | N/A |
| **19.** | Audio/visual recording |  | 9 |
| **20.** | Field notes |  | N/A |
| **21.** | Duration |  | 9 |
| **22.** | Data saturation |  | 11 |
| **23.** | Transcripts returned | The transcripts were not returned to participants but the participant could reach out to the research team with questions and some of the participants  chose to do so. | N/A |
| **Domain 3: analysis and findings** | | |  |
| **Data analysis** | | |  |
| **24.** | Number of data coders | LT coded all data, while UN and LK parts of the data and all were involved in discussing the codes. | 11 |
| **25.** | Description of the coding tree |  | 11 |
| **26.** | Derivation of themes |  | 11 |
| **27.** | Software |  | N/A |
| **28.** | Participant checking | The research team has planned for reporting back to the participants, and presenting the article. | N/A |
| **Reporting** | | |  |
| **29.** | Quotations presented |  | 14-  24 |
| **30.** | Data and findings consistent | No inconsistencies were discovered. | N/A |
| **31.** | Clarity of major themes |  | 13 |
| **32.** | Clarity of minor themes |  | N/A |
